# An Increase in Aspartate Aminotransferase Can Predict Worsening Disease Severity in Japanese Patients with COVID-19

**DOI:** 10.3390/clinpract14040129

**Published:** 2024-08-20

**Authors:** Kengo Matsumoto, Tsutomu Nishida, Dai Nakamatsu, Masashi Yamamoto, Koji Fukui, Osamu Morimura, Kinya Abe, Yukiyoshi Okauchi, Hiromi Iwahashi, Masami Inada

**Affiliations:** 1 Department of Gastroenterology, Toyonaka Municipal Hospital, 4-14-1 Shibahara, Toyonaka 560-8565, Osaka, Japan; tnishida@gh.med.osaka-u.ac.jp (T.N.); dainakamatsu@chp.toyonaka.osaka.jp (D.N.); yamamasa@gh.med.osaka-u.ac.jp (M.Y.); kfukui@chp.toyonaka.osaka.jp (K.F.); umetaniclinic@kvp.biglobe.ne.jp (M.I.); 2 Department of Internal Medicine, Toyonaka Municipal Hospital, Toyonaka 560-8565, Osaka, Japan; morimura@chp.toyonaka.osaka.jp (O.M.); kiabe-alg@umin.net (K.A.); yokauchi@chp.toyonaka.osaka.jp (Y.O.); iwahashi@chp.toyonaka.osaka.jp (H.I.); 3 Diabetes Center, Toyonaka Municipal Hospital, Toyonaka 560-8565, Osaka, Japan

**Keywords:** COVID-19, liver injury, liver test, Japanese patients, SARS-CoV-2

## Abstract

Background: The prognostic significance of liver dysfunction in COVID-19 patients remains unclear. In this study, we investigated the association between liver function test results and severe disease progression in COVID-19 patients. Methods: This retrospective study included consecutive Japanese COVID-19 patients admitted between February 2020 and July 2021. Predictive variables for severe disease progression were identified by multivariate logistic regression analysis. Severe disease-free survival was estimated with the Kaplan–Meier method and Cox regression analysis. Aspartate aminotransferase (AST) was divided into three grades: grade 1, AST < 30 U/L; grade 2, 30 U/L ≤ AST < 60 U/L; and grade 3, AST > 60 U/L. Results: Among 604 symptomatic patients, 141 (23.3%) developed severe disease at a median of 2 days postadmission. The median hospital stay was 10 days, and 43 patients (7.1%) died during hospitalization. Multivariate regression analysis revealed that hypertension, decreased lymphocyte count, and elevated LDH, CRP, and AST levels (grade 2 and grade 3 relative to grade 1) were the significant predictive variables. Severe disease-free survival time was significantly different between the different AST grades (hazard ratio (HR): grade 2 vs. grade 1, 4.07 (95% confidential interval (CI): 2.06–8.03); grade 3 vs. grade 1, 7.66 (95% CI: 3.89–15.1)). Conclusions: The AST level at admission was an independent risk factor for severe disease in hospitalized Japanese patients with COVID-19.

## 1. Introduction

In December 2019, severe acute respiratory syndrome coronavirus 2 (SARS-CoV-2) emerged in Wuhan, China, leading to a global pandemic. The World Health Organization (WHO) named the disease caused by SARS-CoV-2 coronavirus disease 2019 (COVID-19) in February 2020. The disease caused by SARS-2 CoV-2 has been associated with various systemic complications, including liver dysfunction. The disease has spread rapidly, leading to a global pandemic. The first case of COVID-19 in Japan was reported in Kanagawa Prefecture in January 2020. Since then, multiple waves of infections have been observed, caused by different strains, including α, δ, and Omicron strains [1]. Although the seventh wave caused by Omicron strains caused the most cases, relatively few of the cases were severe diseases requiring respiratory care. As of 21 July 2022, the Omicron BA.2 strain has been largely replaced by the BA.5 strain, which has become the dominant strain in Japan [1].

Although COVID-19 has been reported to cause liver dysfunction in both Japanese and international studies, the pathogenesis, treatment, and prognostic significance of hepatic dysfunction in COVID-19 patients remain unclear [2]. Recent reports indicate that more than half of COVID-19 patients exhibit varying levels of liver dysfunction. Elevated aminotransferase levels have been reported in 14–58% of hospitalized patients with COVID-19 [3]. While the degree of aminotransferase level increase is usually mild (<5 times the upper normal limit), severe acute hepatitis and high aminotransferase levels have been documented. However, comprehensive data on the clinical characteristics of liver enzyme elevation and liver failure in Japanese COVID-19 patients are lacking. Although numerous risk factors for severe COVID-19 have been identified, the impact of liver function abnormalities has yet to be investigated owing to confounding factors and the limited number of reports in large Japanese patient cohorts, leaving a critical gap in our understanding. In this study, we aimed to investigate the associations between liver function and disease severity in a cohort of Japanese COVID-19 patients. By quantifying the predictive value of liver enzyme levels, particularly of AST, we aim to improve the clinical management and risk stratification of COVID-19 patients in Japan.

## 2. Patients and Methods

This was a retrospective single-center cohort study of consecutive Japanese patients who were diagnosed with COVID-19 through PCR or antigen methods and admitted to Toyonaka Municipal Hospital between February 2020 and July 2021. This hospital, located in Toyonaka in Osaka Prefecture, serves a population of approximately 400,000 and has been a key hospital for moderate to severe COVID-19 since the first wave of the pandemic [4,5,6,7,8]. Osaka Prefecture, the third most populous prefecture in Japan, established the Osaka Prefectural Inpatient Follow-up Center on 30 March 2020, to coordinate hospitalization based on patient symptoms and triaged patients with COVID-19 according to disease severity.

We assessed the severity and treatment strategy for hospitalized COVID-19 patients based on guidance from the Japanese Ministry of Health, Labor, and Welfare available on the COVID-19 website, which has been available since 7 March 2020. The latest version (8.0) was released in July 2022 (in Japanese) [1]. The severity of COVID-19 was categorized into four groups: mild disease without respiratory symptoms (SpO_2_ ≥ 96%); moderate I disease with breathing difficulties or pneumonia without respiratory failure (93% < SpO_2_ < 96%); moderate II disease requiring oxygen support (SpO_2_ ≤ 93%); and severe disease requiring intensive care unit (ICU) treatment or intubation. This Japanese severity classification is generally equivalent to the WHO severity classification into mild, moderate, severe, and critical disease [9], although there are some differences in the definitions. Treatment strategies have been updated with therapeutic advances. The therapeutic agents used in our hospital were hydroxychloroquine, favipiravir, ciclesonide, and dexamethasone from waves 1 to 3 and favipiravir, dexamethasone, heparin, and remdesivir from waves 4 to 5. We reviewed the clinical characteristics of COVID-19 patients with liver involvement by collecting electronic medical records from our hospital (MegaOak Online Imaging System, NEC, Tokyo, Japan). Clinical and laboratory data were collected from all the patients at hospital admission. We prospectively used medical templates to assess all the hospitalized COVID-19 patients at our infectious disease unit to avoid missing data. Patient height and body weight were obtained from medical interviews to reduce contact with patients. We conducted hepatitis B virus serology and hepatitis C virus antibody tests if they had not been previously conducted. We performed a portable chest radiograph for the initial evaluation of pulmonary complications at admission.

This study was conducted in accordance with the Declaration of Helsinki, and the institutional review board of Toyonaka Municipal Hospital approved this study (3 October 2022). The requirement for informed consent was waived by the same committee using the opt-out method on our hospital website.

In the context of our retrospective study, the data were accessed on 11 December 2022 for research purposes. Throughout the research process, including during and after data collection, we had access to information that could potentially identify individual participants. All the necessary ethical considerations and precautions were followed to maintain confidentiality and comply with applicable data protection regulations.

We investigated several predictive variables associated with the development of severe conditions, including age ≥ 65 years, BMI ≥ 30, smoking status, hypertension status, diabetes status, hyperlipidemia status, chronic kidney disease status, chronic lung disease status, solid cancer status, pregnancy status, lymphocyte count, lactate dehydrogenase (LDH) level, and C-reactive protein (CRP) level. We previously reported that the cutoff values for lymphocyte count, LDH levels, CRP levels, and estimated glomerular filtration rate (eGFR) were 980 count/μL, 309 IU/mL, 2.92 mg/dl, and 68 mL/min, respectively, as predictive risk factors in our hospital cohort [4]. In the present study, we estimated that cutoff values of 1000, 300, 3, and 70 mL/min (CKD) were the predictive risk factors. The FIB-4 index, which evaluates liver fibrosis, was computed using the formula FIB-4 index = age × aspartate aminotransferase (AST)/platelet count (PLT) × √ alanine aminotransferase (ALT) [10]. We also assessed two liver function parameters, aspartate aminotransferase (AST) and alanine aminotransferase (ALT), and categorized patients into three groups at admission based on their AST and ALT levels: those with normal aminotransferase levels (grade 1, AST/ALT < 30 U/L), those with moderately elevated aminotransferase levels (grade 2, 30 U/L ≤ AST/ALT < 60 U/L), and those with highly elevated aminotransferase levels (grade 3, 60 U/L < AST/ALT).

### Statistical Analysis

The continuous variables are presented as medians and interquartile ranges (IQRs), and the categorical variables are summarized as frequencies (%). We used Fisher’s exact test to evaluate differences in the categorical variables and the Kruskal–Wallis test to compare differences in the continuous variables among the three groups. We performed a univariate logistic analysis of the known risk factors for increased severity and stratified the patients into three groups according to their AST and ALT levels to identify significant risk factors. When both AST and ALT levels were identified as significant factors in the univariate analysis, we selected statistically significant factors with higher odds ratios due to confounding factors. We used logistic multivariate analysis with the extracted factors to examine the effects of liver dysfunction test values on illness severity. We estimated the severe disease-free survival time from admission using the Kaplan–Meier method and evaluated hazard ratios (HRs) among the three AST groups using Cox regression analysis.

All the calculated p values were two-tailed, with a *p* value < 0.05 considered to indicate statistical significance. We performed all the statistical analyses using the JMP statistical software (ver. 16.20; SAS Institute Inc., Cary, NC, USA).

## 3. Results

**Figure 1:**
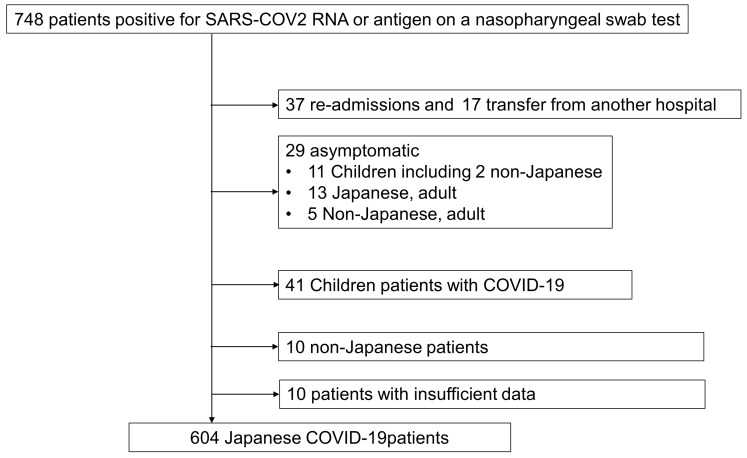
Flow chart of patient enrollment. COVID-19. We enrolled 748 COVID-19 patients during the study period, and 604 patients were included in the final analysis.

During the study period, 748 COVID-19 patients were enrolled. Of these, 37 readmitted patients, 17 patients transferred from another hospital, 29 asymptomatic patients, 41 patients younger than 16 years, and 10 non-Japanese patients were excluded, as were 10 patients with insufficient data. Ultimately, 604 patients were included in the final analysis (Figure 1).

The clinical characteristics of the enrolled COVID-19 patients are summarized in Table 1. The median age was 62 years (IQR, 47–78), and 335 patients (55.5%) were men. The median BMI was 23.9 (IQR 21.0, 26.9), and 34.9% and 34.9% of the patients had a history of alcohol consumption and smoking, respectively. Other comorbidities included hypertension in 270 (44.1%) patients, cardiac disease in 103 (17.2%), diabetes in 168 (27.9%), hyperlipidemia in 133 (23.3%), and pregnancy in 23 (3.8%). Regarding medications, 148 (24.5%) patients were taking angiotensin-converting enzyme inhibitors (ACEis)/angiotensin receptor blockers (ARBs), 190 (31.6%) were taking calcium blockers, and 110 (18.3%) were taking statins.

The median time from onset to admission was 6 days (IQR: 4–9 days). On admission, 92.7% of the patients had fever, 48% had fatigue, 77.3% had respiratory symptoms, and 75.6% had pneumonia. Twenty-five patients (4.1%) were diagnosed with severe disease on admission, and 141 (23.3%) developed severe disease within a median of 2 days (IQR 1, 5) of hospitalization. The median length of hospital stay was 10 days (IQR 7, 15), and 43 (7.1%) patients died during hospitalization (Table 2).

### 3.1. Liver Function Tests at Admission Can Be Used to Predict Worsening Disease Severity in Japanese Patients with COVID-19

The median levels of AST and ALT at admission were 32 IU/mL (23, 49) and 24 IU/mL (15, 40), respectively. The peak AST and ALT levels during hospitalization were 41 IU/mL (27, 64) and 39 IU/mL (21, 73), respectively. The median alkaline phosphatase (ALP), γ-glutamyl transferase (GGT), and total bilirubin levels and FIB-4 index score at admission were 168 IU/mL (74, 216), 35 IU/mL (18, 69), 0.55 mg/dL (0.4, 0.71), and 2.19 (1.32, 3.60), respectively.

### 3.2. Univariate Logistic Analysis and Univariate Cox Proportional Hazards Analysis of Risk Factors for Progression to Severe COVID-19

We conducted a univariate logistic analysis of the 13 established risk factors [1] and AST and ALT levels to evaluate their associations with progression to severe disease among 579 patients. The patients with severe disease at admission were excluded from the analysis. Older age (OR: 2.1, *p* = 0.0002), hypertension (OR: 2.94, *p* < 0.0001), diabetes mellitus (OR: 1.84, *p* = 0.0003), a decreased eGFR (OR: 2.38, *p* < 0.0001), a decreased lymphocyte count (OR: 3.03, *p* < 0.0001), an elevated LDH level (OR: 3.88, *p* < 0.0001), an elevated CRP level (OR: 3.29, *p* < 0.0001), and higher AST and ALT grades (ORs for grade 3 to grade 1 and grade 2 to grade 1: 5.13 and 2.36, *p* < 0.0001 and *p* = 0.0002, respectively) were significantly associated with progression to severe disease (Table 3). Among these factors, AST grade had a significantly greater OR than did ALT grade; thus, AST grade was selected as a risk factor for the subsequent multivariate regression analysis. Multivariate regression analysis of 14 variables (excluding ALT grade) revealed hypertension (OR: 2.24, *p* = 0.0026), a decreased lymphocyte count (OR: 2.72, *p* < 0.0001), an elevated LDH level (OR: 1.87, *p* = 0.002), an elevated CRP level (OR: 1.96, *p* = 0.016), and an elevated AST level (ORs of 1.83 for grade 2 and 3.35 for grade 1, *p* = 0.0038, and *p* = 0. 0009) were significantly associated with progression to severe disease. Among the significant risk factors at admission, the AST > 60 group had the highest OR for predicting progression to severe disease compared to the AST < 30 group.

### 3.3. Clinical Course of COVID-19 Patients Based on AST Grade

**Figure 2:**
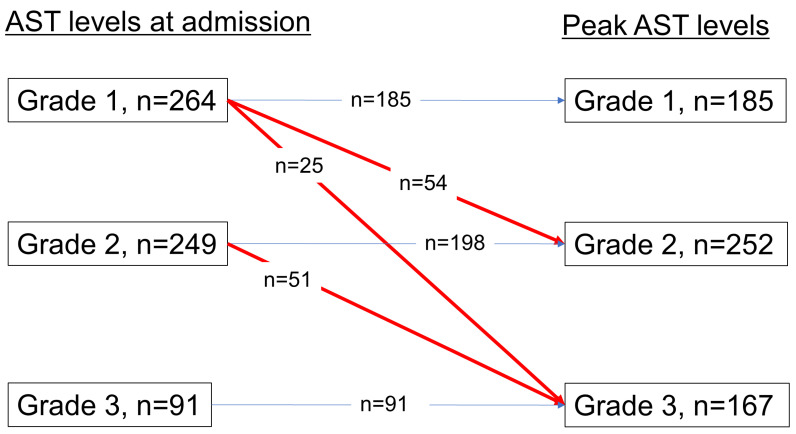
AST levels on admission and peak AST levels. During the clinical course, 79 patients with grade 1 ASTs were upgraded to a higher AST grade, and 51 patients with grade 2 ASTs were upgraded to grade 3, although the AST grade did not change in 383 patients with grades 1 and 2.

**Figure 3:**
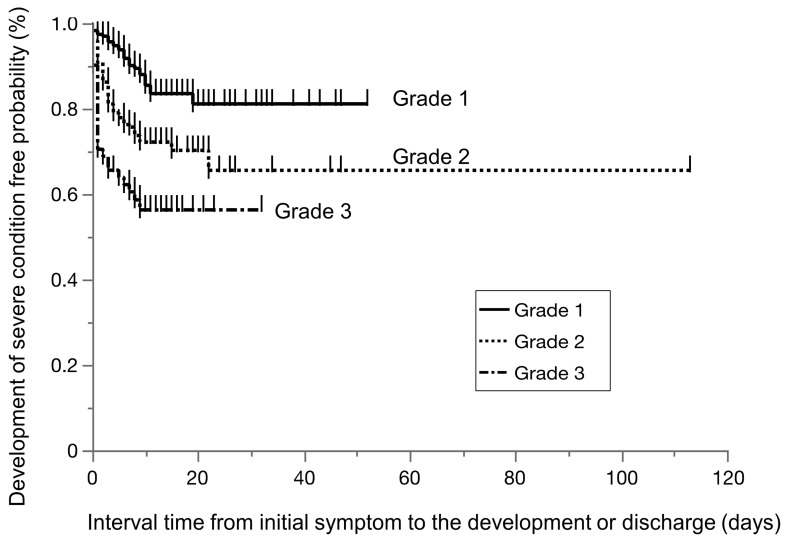
The severe disease-free survival times in the three grade groups. The three groups were significantly separated in parallel according to the severity of AST.

Table 4 compares the clinical characteristics of COVID-19 patients with AST levels of 1, 2, and 3 on admission. Among the 579 patients, 264 had grade 1 AST, 249 had grade 2 AST, and 91 had grade 3 AST on admission. Grade 1 restraint was significantly more prevalent in females and less prevalent in patients with a high BMI than in those with other BMIs. During the clinical course, 79 patients with grade 1 ASTs (30%) had worsened ASTs and were upgraded to higher ASTs (grade 2, n = 54; grade 3, n = 25), and 51 patients (20.5%) with grade 2 ASTs were upgraded to grade 3, whereas 383 patients with grades 1 and 2 (76%) did not have their ASTs changed (Figure 2). The rates of progression to severe disease were 13.4% (35/259) for grade 1 AST, 26.5% (66/236) for grade 2 AST, and 44.0% (40/84) for grade 3 AST (*p* < 0.0001). The severe disease-free survival times in the three grade groups were significantly separated in parallel according to the AST severity (Figure 3, HR of grade 2 to grade 1: 4.07 (95% CI: 2.06–8.03, *p* < 0.0001), HR of grade 3 to grade 1: 7.66 (95% CI: 3.89–15.1, *p* < 0.0001)).

### 3.4. Patients with Underlying Liver Disease

In the present study, we investigated patients with underlying liver disease. Overall, three patients were found to have positive hepatitis B surface (HBs) antigen tests, while 18 had positive hepatitis C (HCV) antibody tests. None of the patients had autoimmune hepatitis or primary biliary cholangitis during the study period, as indicated in Table 5. Notably, one out of three patients who had a positive HBs antigen test was found to be coinfected with HCV. Overall, 20 patients tested positive for viral hepatitis. Among these patients, six patients (30%) developed severe COVID-19. The median time from admission to progression to severe disease was three days (IQR: 0–3.5 days). The median AST and ALT levels were 29 IU/mL (IQR 23, 39) and 18 IU/mL (IQR 14, 32), respectively. Furthermore, the median peak AST and ALT levels during hospitalization were 40 IU/mL (IQR 26, 97) and 39 IU/mL (IQR 20, 71), respectively. The AST grade at the time of admission was 1 for thirteen patients, 2 for four patients, and 3 for three patients. During the clinical course, six patients (30.0%) experienced an increase in the AST grade, but 14 patients did not experience any change in the AST grade.

## 4. Discussion

Blood tests provide important information on the condition of patients with COVID-19 and aid in their prognosis. Therefore, blood tests are recommended for hospitalized COVID-19 patients at risk of severe disease [1]. Several studies on biomarkers for disease progression have been conducted both in Japan and internationally [11]. Our study demonstrates that elevated AST at admission is a significant independent risk factor for severe disease in hospitalized Japanese COVID-19 patients. This finding suggests that liver function tests should be considered in the clinical assessment of COVID-19 patients to better predict disease progression. The application of these markers is expected to enhance the quality of medical care and optimize the utilization of medical resources. Specifically, our multivariate analysis incorporating the established risk factors revealed that elevated AST level at admission was an independent and significant risk factor for severe disease in hospitalized Japanese COVID-19 patients. Among the COVID-19 patients who presented with AST ≥ 60 IU/mL on admission, 44.0% progressed to severe disease, which was 1.8 times greater than the rate in the entire cohort.

Regarding the impact of underlying liver disease, no significant findings were obtained due to the low number of patients with viral hepatitis or autoimmune diseases in this cohort. Owing to the constraints of minimizing contact with patients, abdominal ultrasound (US) was not performed; thus, we were unable to verify previous data or identify patients with alcoholic liver disease or nonalcoholic fatty liver disease [12]. Although a reduction in PLT is indicative of the severity of liver fibrosis [13], it was challenging to determine whether the patients had underlying liver disease based on their PLT in this study, as PLT decreases in patients with COVID-19 infection due to inflammation and intravascular coagulation disorders [14].

In previous reports, hypertension, diabetes mellitus, and hyperlipidemia status have been identified as risk factors for severe COVID-19 status [5,15]. In the present study, hypertension and diabetes mellitus status were significantly associated with disease severity in univariate logistic analysis. In univariate logistic analysis, when the patients who met two or more of the criteria for hypertension, dyslipidemia, and diabetes were examined, they were also shown to be at a significantly greater risk of severe disease status. However, the odds ratio for that association was not higher than the odds ratio for the risk of severe disease status in patients with hypertension alone. The same was observed for the patients who met the criteria for metabolic syndrome, where the odds ratio was also not higher than that observed in patients with hypertension alone. Ferritin has been used both as an inflammatory marker and an indicator of chronic hepatitis, and an association between ferritin levels and the risk of COVID-19 severity has been reported [16]. However, many patients in our cohort had missing ferritin measurements, making a comprehensive analysis difficult. In the subset of 523 patients for whom data were available, a cut-off value of 500 ng/mL indicated a 3.38-fold increase in the probability of severe disease for patients with higher ferritin levels. However, as ferritin levels are strongly associated with inflammation, it is difficult to infer the presence of chronic liver disease solely from the ferritin levels on admission.

The liver may be vulnerable to SARS-CoV-2 infection due to the presence of angiotensin-converting enzyme 2 (ACE2) receptors on biliary and hepatic epithelial cells [17]. The virus enters and harms target organs by binding to ACE2 receptors [18,19]. Autopsy results have confirmed the presence of viral RNA in liver tissue [20], indicating the possibility of direct hepatocellular damage caused by SARS-CoV-2. In our study, 97 patients received ARBs, 9 received ACEi, and 45% had hypertension. Among the 105 patients who were treated with ARBs and/or ACE inhibitors, 35.2% (37/105) progressed to severe disease, which was significantly greater than the percentage of patients (20.5%; 82/401) who did not receive these medications (*p* = 0.00027). In addition, AST severity was significantly greater in the ARB and/or ACE inhibitor users than in nonusers (grade 1 in 36.5% of the users and 50.1% of the nonusers, grade 2 in 48.2% of the users and 38.0% of the nonusers, and grade 3 in 15.5% of the users and 11.9% of the nonusers; *p* = 0.0478). Our findings suggested that ARB/ACE inhibitor use may upregulate ACE2 receptor expression in biliary and hepatic epithelial cells, which could lead to liver dysfunction in ARB/ACE inhibitor users. However, the impact of hypertension as a confounding factor could not be determined. Furthermore, liver dysfunction in COVID-19 patients may also be attributed to inflammation, cytokine storms, the use of therapeutic drugs, and hypoxemia associated with respiratory failure.

This study described the first to fifth waves of the COVID-19 outbreak in Japan, during which different mutant strains were prevalent, leading to varying infectivity and symptoms. Since the number of available drugs increased from the fourth period, we classified patients into two groups: those in the earlier period up to the third period and those in the latter period after the fourth period. We investigated differences in the background and frequency of liver injury between these two groups. The frequency of liver injury at presentation was greater in the latter period (grade 1 (48.8%, 35.6%), grade 2 (39.9%, 43.4%), and grade 3 (11.3%, 21.0%)). However, the frequency of AST level upgrades was not significantly different between the two groups (21.8%, 21.0%, *p* = 0.81). Although there was no significant difference in the severity of admission between the two groups, the frequency of progression to severe disease was significantly greater in the latter group (20.0%, 28.8% *p* = 0.013) (Table 6). Although it was challenging to assess this due to differences in hospitalization criteria during each wave, it was suggested that the variation in viral strains might have an impact on liver damage.

With the spread of COVID-19, the existing drugs have been repurposed, and several treatments have been approved, but liver damage has been reported with COVID-19 treatments [21,22,23,24,25]. Such treatments have also been reported to induce liver injury [26]. At our hospital, chloroquine, favipiravir, ciclesonide, and dexamethasone were used from the first to the third wave, and favipiravir, decadron, heparin, and remdesivir were used from the fourth wave to the fifth wave. The impact of each drug on liver dysfunction was studied. Univariate logistic analysis of the six drugs, disease progression to severe COVID-19, and worsening AST levels during hospitalization indicated that the use of favipiravir, ciclesonide, and remdesivir was a significant factor in exacerbating COVID-19. Hepatic injury exacerbation after hospitalization was influenced by drug use as well as by worsening COVID-19 symptoms; however, drawing conclusions is challenging due to several confounding factors (Table 7).

The study has several limitations. First, it was a retrospective, single-center study, which may limit the generalizability of the findings. Second, minimal contact with patients during the COVID-19 lockdown may have led to an inadequate assessment of previous liver function abnormalities. Additionally, distinguishing between hepatocellular and cholestatic liver injury is necessary for a more detailed description of liver injury, but many patients in our cohort had deficient ALP levels, making this analysis difficult in the present study. Third, differences in viral strains were not examined. In Japan, there were four pandemic waves from 2020 to July 2021, with the α and δ subtypes prevalent. Although the importance of variant-specific outcomes has been reported, it was difficult to distinguish which variant each patient had based on our laboratory tests. Fourth, the relationship between drug use and liver injury was examined, but the patients were administered different drugs, making it difficult to draw conclusions about the effects of the drugs. Therefore, the involvement of drugs used during hospitalization has not been fully investigated. The use of antidiabetic drugs and statins may be associated with liver dysfunction, while an association between their use and COVID-19 severity has also been reported [4,27,28]. This was difficult to examine in the present study due to the limited data on antidiabetic drug use during hospitalization and the use of the attending physician’s discretion to decide whether to continue the use of medication. The study was observational and although it was possible to identify an association between transaminases and patient outcomes, it was not possible to establish a causal relationship.

In conclusion, our study demonstrated that AST levels at admission were a significant independent risk factor for severe disease in hospitalized Japanese patients with COVID-19. By incorporating liver function tests into routine clinical assessments, healthcare providers can better predict disease progression and improve patient outcomes.

## Figures and Tables

**Table 1:** Characteristics of symptomatic patients with confirmed COVID-19 on admission.

Characteristics	Patients with COVID-19, n = 604
Age, median (IQR)	62 (47, 78)
Sex, male n (%)	335 (55.5)
Body mass index, median (IQR)	23.9 (21.0, 26.9)
Smoking history (none/past/current)	393/33/178
Drinking history, yes n (%)	211 (34.9)
Days from the onset of symptoms to admission, median (IQR)	6 (4, 9)
Laboratory data on admission	
WBC, median (IQR) count/μL	5100 (4050, 6800)
Lymphocyte, median (IQR) (count/μL)	920 (672, 1240)
Neutrophilia, median (IQR) (count/μL)	3699 (2663, 5252)
Hemoglobin, median (IQR) (g/dL)	13.7 (12.3, 14.8)
Platelet count, median (IQR) (10^9^/L)	18.4 (14.7, 23.0)
AST, median (IQR) (IU/L)	32 (23, 49)
ALT, median (IQR) (IU/L)	24 (15, 40)
ALP, median (IQR) (IU/L) ※1	168 (74, 216)
γGTP, median (IQR) (IU/L) ※2	35 (18, 69)
T-bil, median (IQR) (mg/dL)	0.55 (0.4, 0.71)
FIB-4 index, median (IQR)	2.19 (1.32, 3.60)
Cr, median (IQR) (mg/dL)	0.81 (0.63, 1.10)
BUN, median (IQR) (mg/dL)	14 (11, 20)
LDH, median (IQR) (IU/L)	262 (201, 356)
CRP, median (IQR) (mg/dL)	3.6 (0.78, 7.9)
eGFR (mL/min/1.73m^2^), median (IQR)	69.1 (50.7, 87.7)
HbA1c, median, median (IQR) (%)	6 (5.7, 6.6)
Casual blood glucose, median (IQR) (mg/dL)	115 (100, 140)
T-Chol, median (IQR) (mg/dL)	164 (142, 189)
Comorbidities	
Hypertension, n (%)	270 (44.1)
Cardiovascular diseases, n (%)	103 (17.2)
Chronic obstructive pulmonary disease, n (%)	32 (5.4)
Asthma, n (%)	49 (8.2)
Diabetes mellitus, n (%)	168 (27.9)
Hyperlipidemia, n (%)	133 (23.3)
Chronic kidney disease, n (%)	71 (11.9)
Hemodialysis, n (%)	36 (6.1)
Solid cancer, n (%) ※3	48 (8.0)
Pregnancy, n (%)	23 (3.8)
Concomitant liver disease	
HBs Ag positive, n (%)	3 (0.53)
HCV Ab positive n (%)	18 (3.2)
AIH or PBC, n (%)	0 (0)
Use of medication for comorbidities	
ACEi/ARB, n (%)	148 (24.5)
Calcium blocker, n (%)	190 (31.6)
Statin, n (%)	110 (18.3)
PPI, n (%)	133 (22.2)

※1: ALP levels were deficient in 339 cases; ※2: γGTP levels were deficient in 152 cases; ※3: 6 patients undergoing chemotherapy. IQR: interquartile range; HBs Ag: hepatitis B antigen; HCV Ab; hepatitis C antibody; AIH: autoimmune hepatitis; LDH: lactate dehydrogenase; CRP: C-reactive protein; AST: aspartate aminotransferase; ALT: alanine aminotransferase; Cr: creatinine; BUN: blood urea nitrogen; eGFR: estimated glomerular filtration rate; ACEi: angiotensin-converting enzyme inhibitor; ARB: angiotensin receptor blocker; PBC: primary biliary cholangitis; PPI: proton pump inhibitor.

**Table 2:** Initial presentation on admission, treatment, and clinical course.

Initial Presentation	
Fever, n (%)	559 (92.7)
Fatigue, n (%)	206 (48)
Respiratory-related symptoms, n (%)	468 (77.3)
Pneumonia, n (%)	456 (75.6)
Headache, n (%)	55 (12.3)
New loss of taste or smell, n (%)	117 (23.2)
Days from onset of symptoms to admission, median (IQR)	5 (3, 8)
Severity of COVID-19 on admission	
Mild to moderate symptoms, n (%)	579 (95.9)
Severe symptoms, n (%)	25 (4.1)
Progression to severe disease, n (%)	141 (23.3)
Treatment	
Required oxygen, n (%)	375 (62.1)
Medication for COVID-19	
Ciclesonide, n (%)	156 (31.2)
Hydroxychloroquine, n (%)	16 (2.6)
Favipiravir, n (%)	291 (48.1)
Heparin, n (%)	52 (8.6)
Remdesivir, n (%)	35 (5.8)
Dexamethasone, n (%)	305 (50.5)
Clinical course	
Peak AST, median (IQR) (IU/L)	41 (27, 64)
Peak ALT, median (IQR) (IU/L)	39 (21, 73)
Length of hospital stay, median (IQR) (days)	10 (7, 15)
Time from admission to aggravation (IQR) (days)	2 (1, 5)
Required mechanical ventilatory support, n (%)	125 (24.3)
Mortality, n (%)	43 (7.1)

IQR: interquartile range; AST: aspartate aminotransferase; ALT: alanine aminotransferase.

**Table 3:** Univariate logistic analysis and multivariate regression analysis of risk factors for progression to critical COVID-19.

	Univariate Logistic Analysis			Multivariate Logistic Analysis		
Variables	Odds Ratio	95% CI	*p* Value	Odds Ratio	95% CI	*p* Value
Aged 65 years and over, yes	2.1	1.43–3.09	0.0002	1.32	0.78–2.23	0.29
BMI 30 and over, yes	1.51	0.87–2.62	0.14	1.39	0.72–1.88	0.32
Smoking history, yes	1.33	0.88–2.03	0.17	1.05	0.64–1.71	0.84
Hypertension, yes	2.94	1.99–4.37	<0.0001	2.24	1.32–3.78	0.0026
Diabetes mellitus, yes	1.84	1.23–2.75	0.003	1.25	0.76–2.07	0.38
Hyperlipidemia, yes	1.73	1.12–2.67	0.01	1.12	0.66–1.88	0.67
Chronic kidney disease (eGFR < 70), yes	2.38	1.60–3.54	<0.0001	1.52	0.92–2.50	0.10
Chronic lung disease, yes	1.55	0.71–3.36	0.27	1.28	0.51–3.21	0.60
Solid cancer, yes	1.00	0.50–2.02	0.99	0.97	0.42–2.20	0.93
Pregnancy, yes	3.30	0.76–14.3	0.11	2.01	0.41–9.91	0.39
Lymphocyte count < 1000, yes	3.03	1.98–4.67	<0.0001	2.72	1.63–4.56	<0.0001
LDH ≥ 300, yes	3.42	2.31–5.06	<0.0001	1.87	1.10–3.16	0.020
CRP ≥ 3, yes	3.29	2.15–5.02	<0.0001	1.96	1.13–3.40	0.016
Elevated AST						
Grade 1, AST < 30 IUL	1					
Grade 2, 30 < AST < 60	2.36	1.50–3.71	0.0002	1.83	1.04–3.24	0.038
Grade 3, AST > 60	5.13	2.96–8.86	<0.0001	3.35	1.64–6.81	0.0009
Elevated ALT						
Grade 1, ALT < 30 IU/L	1					
Grade 2, 30 < ALT < 60	1.65	1.08–2.52	0.02			
Grade 3, ALT > 60		1.07–3.27	0.02			

CI: confidence interval; BMI: body mass index, LDH: lactate dehydrogenase; CRP: C-reactive protein; eGFR: estimated glomerular filtration rate; AST: aspartate aminotransferase; ALT: alanine aminotransferase.

**Table 4:** Comparison of three groups according to AST level on admission.

Characteristics	Grade 1 Normal AST n = 264	Grade 2 30 ≤ AST < 60 n = 249	Grade 3 ALT > 60 n = 91	*p* Value
Age, median (IQR)	60 (37, 80)	63 (50, 78)	64 (53, 73)	0.1200
Men Sex, n (%)	110 (41.7)	156 (62.65)	69 (75.8)	<0.0001
Body mass index > 30, n (%)	21 (8.6)	37 (15.7)	14 (15.9)	0.0400
Smoking history, yes, n (%)	77 (29.2)	91 (36.6)	43 (47.3)	<0.0001
Severity of COVID-19				
Mild to moderate symptom, n (%)	259 (98.1%)	236 (94.8%)	84 (92.3%)	0.03
Severe symptom, n (%)	5 (1.9%)	13 (5.2%)	7 (7.69%)	0.03
Progression to severe disease, n (%)	35 (13.3)	66 (26.5)	40 (44.0)	<0.0001
Diabetes mellitus, n (%)	58 (22.1)	82 (32.9)	28 (30.8)	0.0200
Hyperlipidemia, n (%)	34 (13.4)	72 (30.8)	27 (32.9)	<0.0001
Chronic kidney disease, n (%)	44 (16.7)	21 (8.6)	6 (6.6)	0.005
Hemodialysis, n (%)	28 (10.9)	7 (2.85)	1 (1.16)	0.0001
Solid cancer, n (%)	17 (6.5)	24 (9.8)	7 (7.7)	0.38
Pregnancy, n (%)	20 (7.6)	3 (1.2)	0 (0.0)	<0.0001
Concomitant liver disease				
HBs Ag positive, n (%)	2 (0.8)	1 (0.44)	0 (0)	0.76
HCV Ab positive n (%)	11 (4.4)	4 (1.8)	3 (3.8)	0.45
AIH or PBC, n (%)	0	0	0	
Use of medication for comorbidities				
ACEi/ARB, n (%)	53 (20.1)	71 (28.5)	24 (26.4)	0.07
Calcium blocker, n (%)	74 (28.0)	89 (36.2)	27 (29.7)	0.13
Statin, n (%)	35 (13.3)	58 (23.6)	17 (18.9)	0.02
PPI, n (%)	55 (20.8)	59 (24.0)	19 (20.9)	0.66
Laboratory data				
Lymphocytes, median (IQR) (count/μL)	1011 (724, 1336)	890 (637, 1113)	856 (616, 1145)	0.0001
LDH, median (IQR) (IU/L)	205 (172, 253)	298 (239, 368)	422 (342, 553)	<0.0001
CRP, median (IQR) (mg/dL)	1.41 (0.37, 4.84)	4.62 (1.96, 9.25)	6.69 (3.94, 12.64)	<0.0001
eGFR (mL/min/1.73 m^2^), median (IQR)	71.6 (48.8, 90.7)	65.6 (50.7, 81.3)	70 (52.8, 85.7)	0.0900
HbA1c, median, (IQR) (%)	5.8 (5.5, 6.3)	6.2 (5.8, 6.7)	6.3 (5.9, 6,9)	<0.0001
Platelets, median (IQR) (10^9^/L)	19 (15.1, 23.7)	17.9 (14.5, 22)	18.3 (14.7, 22.8)	0.24

IQR: interquartile range; HBs Ag: hepatitis B antigen; HCV Ab: hepatitis C antibody; AIH: autoimmune hepatitis; PBC: primary biliary cholangitis; ACEi: angiotensin-converting enzyme inhibitor; ARB: angiotensin receptor blocker; PPI: proton pump inhibitor; LDH: lactate dehydrogenase; CRP: C-reactive protein; eGFR: estimated glomerular filtration rate.

**Table 5:** Clinical course and liver function tests in patients with underlying liver disease.

Characteristic	Patients with Liver Disease, n = 20
HBs Ag positive, n	3
HCV infection (Current/preexisting), n	9/9
Age, median (IQR)	70 (48.5, 78)
Sex, male n (%)	13 (65)
Laboratory data on admission	
AST, median (IQR) (IU/L)	29 (23, 39)
AST levels on admission n (Grade 1/Grade 2/Grade 3)	13/4/3
ALT, median (IQR) (IU/L)	18 (14, 32)
Platelet count, median (IQR) (10^9^/L)	13.55 (11.83, 17.33)
Fib-4 index, median (IQR)	2.81 (1.71, 4.38)
Clinical course	
Peak AST, median (IQR) (IU/L)	40 (26, 97)
Peak AST levels n (Grade 1/Grade 2/Grade 3)	8/6/6
Peak ALT, median (IQR) (IU/L)	39 (20, 71)
Progression to severe disease, n (%)	6 (30)
Time from admission to severe disease (IQR) (days)	3 (0, 3.5)
Mortality, n (%)	3 (15%)

IQR: interquartile range, AST: aspartate aminotransferase; ALT: alanine aminotransferase.

**Table 6:** Between-group comparison according to the period of illness.

Characteristics	Within the Period from the First to Third Wave n = 371	Within the Period from the Fourth to Fifth Wave n = 233	*p* Value
Age, median (IQR)	57 (43,71)	69 (49, 81)	<0.0001
Male sex, n (%)	200 (59.7%)	135 (57.9)	0.33
Body mass index, median (IQR)	24.3 (21.1, 27.2)	23.5 (20.8, 26.7)	0.17
Days from onset of symptoms to admission, median (IQR)	7 (4, 9)	6 (4, 10)	0.86
Severity of COVID-19 on admission			
Mild to moderate symptoms, n (%)	359 (96.8)	220 (94.4)	0.16
Severe symptoms, n (%)	12 (3.2)	13 (5.6)	0.16
Progression to severe disease, n (%)	74 (20.0)	67 (28.8)	0.013
Medications			
hydroxychloroquine, n (%)	16 (4.3)	0 (0)	0.0001
favipiravir, n (%)	185 (49.9	106 (45.5)	0.83
ciclesonide, n (%)	154 (41.5)	2 (0.86)	<0.0001
heparin, n (%)	7 (1.9)	45 (19.3)	<0.0001
dexamethasone, n (%)	153 (41.2)	152 (65.2)	<0.0001
remdesivir, n (%)	0 (0)	35 (15.0)	<0.0001
AST levels at admission, n (%)			
Grade 1, AST < 30 U/L	181 (48.8)	83 (35.6)	0.0014
Grade 2, 30 < AST < 60	148 (39.9)	101 (43.4)	0.4
Grade 3, 60 < AST	42 (11.3)	49 (21.0)	0.0013
Peak AST levels			
Grade 1, AST < 30 U./L	128 (34.5)	5 7 (24.5)	0.009
Grade 2, 30 < AST < 60	158 (42.6)	94 (40.3)	0.58
Grade 3, 60 < AST	85 (22.9)	82 (35.2)	0.0011
Increased AST grade, n (%)	81 (21.8)	49 (21.0)	0.81

IQR: interquartile range; AST: aspartate aminotransferase; ALT: alanine aminotransferase.

**Table 7:** Univariate logistic analysis of risk factors for increasing AST levels with drug use.

	Univariate Logistic Analysis		
Drugs	Odds Ratio	95% CI	*p* Value
hydroxychloroquine, yes	1.19	0.34–4.25	0.78
favipiravir, yes	2.92	1.93–4.41	<0.0001
ciclesonide, yes	1.73	1.14–2.63	0.01
heparin, yes	1.24	0.64–2.40	0.52
dexamethasone, yes	2.18	1.46–3.27	0.0002
remdesivir, yes	1.50	0.69–3.20	0.30
Progression to severe symptoms, yes	1.81	1.17–2.78	0.0068

CI: confidence interval.

## Data Availability

Data supporting the findings of this study are available upon request from the corresponding author, Matsumoto K. The data are not publicly available due to restrictions (e.g., they contain information that could compromise the privacy of the research participants).
